# Elimination of radioactivity after intratracheal instillation of tritiated 3,4-benzopyrene in hamsters.

**DOI:** 10.1038/bjc.1969.16

**Published:** 1969-03

**Authors:** L. N. Pylev, F. J. Roe, G. P. Warwick


### ELIMINATION OF RADIOACTIVITY AFTER INTRATRACHEAL INSTILLATION OF TRITIATED 3,4-BENZOPYRENE IN HAMSTERS

L. N. PYLEV, F. J. C. ROE AND G. P. WARWICK

*From the Laboratory of Cancer Prevention, Institute of Experimental and Clinical Oncology AM.S. U.S.S.R., 6 Kashirskoye Shosse, Moscow, U.S.S.R., and the Chester Beatty Research Institute, Fulham Road, London, S.W.3, England*

Received for publication October 29, 1968

A WIDE variety of inhalable substances are known to contribute to the total toll of deaths from lung cancer in man. These include tobacco smoke, air pollutants, nickel carbonyl, arsenic-containing and chromium-containing compounds, asbestos dust, and various radioactive dusts. Relatively little is known of the mechanisms by which these agents act to induce lung cancer either singly or in combination (see Roe and Walters, 1965 for review).

One requirement for the detailed study of lung cancer aetiology is the availability of a model laboratory animal system in which it is possible to induce lung cancer in relatively high incidence. As described in previous publications an experimental model for the study of the induction of lung cancer has been developed (Pylev, 1961, 1962, 1963 and 1964). 9,10-Dimethyl-1,2-benzanthracene (DMBA) or 3,4-benzopyrene (BP) mixed with India ink powder as a suspension in casein solution was injected intratracheally in rats. In the experiments with DMBA, lung tumours (squamous cell carcinomas and adenocarcinomas) were induced in almost 30% of rats (Pylev, 1961, 1962), while in the experiments with BP lung tumours (mostly squamous cell carcinomas) were found in 67% of rats (Pylev, 1962, 1964). Other methods of exposure have always led to a lower incidence of tumours (Niskanen, 1949; Kuschner, Laskin, Cristofano and Nelson, 1957; Della Porta, Kolb and Schubik, 1958; Blacklock, 1957, 1961). It has been suggested that the higher incidence of lung cancer seen in the experiments of Pylev might be attributable to prolongation of the period during which the carcinogen remains in contact with lung tissue. Particles of India ink and carbon black, which are less readily removed from the lungs than substances in solution, are able to adsorb carcinogens and thereby delay their elimination from the lungs.

It has been shown that the amount of the carcinogen which can be retained in the lungs depends on the size of the adsorbent particles and on their adsorbtive capacity. Thus BP injected with thermal carbon black (particle size 3047 Å) was eliminated from rat lung more quickly than BP injected alone, but the same compound injected with channel carbon black (particle size 128 Å) was eliminated more slowly than BP alone (Pylev, 1962, 1967; Shabad, Pylev and Kolesnichenko, 1964). Saffiotti and his colleagues (1964) obtained very similar results with haematite. Harington (1962) and Roe, Walters and Harington (1966) have shown that commercial asbestos contains carcinogenic polycyclic hydrocarbons. Their presence is partly due to contamination from jute bags in which it is customarily stored and transported. Carcinogen-containing mineral oils are used in the processing of jute, and these are adsorbed by the asbestos fibres during storage (Harington, 1965; Harington and Roe, 1965). The possibility that these adsorbed carcinogen are wholly or partly responsible for the carcinogenicity of inhaled asbestos has to be considered. In this connection a recent report by Selikoff and his colleagues (1968) is of special interest. They found that the risk of death from bronchogenic carcinoma among asbestos workers was only excessive if they were also smokers.

These facts stimulated us to undertake a comparison of the rate of elimination of [$^3$H]3,4-benzopyrene ([$^3$H]BP) from animals subject to a single intratracheal injection of the labelled carcinogen alone, and from animals injected with the labelled carcinogen plus either asbestos or carbon black.

In the present experiments hamsters were used since it has been observed (Saffiotti and his colleagues, 1964) that spontaneous lung tumours are uncommon in these animals, while they are quite susceptible to lung tumour induction under experimental conditions (Herrold and Dunham, 1962, 1963; Saffiotti *et al.*, 1964). Hamsters differ from rats in lung structure and in pulmonary mucus secretion, but have the advantage over rats in being less prone to spontaneous pulmonary disease (Roe and Walters, 1965).

### MATERIALS AND METHODS

Female hamsters, weighing 80–150 g., of the cream-coated or golden varieties were used, depending on availability, in different tests. They were housed in plastic cages and given Oxoid Breeding Diet and water *ad libitum.*

Tritium labelled 3,4-benzopyrene ([$^3$H]BP) (specific activity 529 mCi/mM) was obtained from the Radiochemical Centre (Amersham, England). Cold BP was obtained from L. Light and Co. and purified in the Chemistry Department of the Chester Beatty Research Institute, on a column of activated aluminium oxide. This was used to dilute the radioactive material to the required specific radioactivity.

Assays of radioactivity were carried out by the use of a Packard Tri Carb Liquid Scintillation Spectrometer Model 3375 and counting efficiency was determined by the channels ratio method.

Furnace carbon black, with 90% particle size 26–160 m$\mu$, and crocidolite from the UICC standard samples were used (Timbrell, Gilson and Webster, 1968).

Aminosol vitrum 10%, which contains 10% amino acids and low-molecular peptides obtained by enzymatic hydrolysis of animal proteins in water, was obtained from Vitrum Company (Stockholm, Sweden) and was used as a vehicle. A small amount (less than 0·02 ml per 4 ml. of aminosol vitrum) of Tween 80 was added and a fine suspension prepared by use of a magnetic stirrer. Stirring was continued until the injections had been completed.

Hamsters were anaesthetized with ether. 0·2 ml. of suspension was introduced into the trachea via the larynx through a blunt metal needle attached to a tuberculin syringe. After injection, animals were kept for a few seconds in a vertical position to prevent any of the suspension flowing out.

Hamsters were killed with ether in the first experiment and by decapitation in the second and the third experiments.

Lungs, liver and kidneys were removed, quickly weighed and placed in cold ethanol (10 ml. for lungs and kidneys, and 30 ml. for liver.) Samples were homogenized in an Ultra-Torrax homogenizer for 3–4 min. at O° C. The homogenate (1 ml.) was mixed with tetraethylammonium hydroxide (TEH) (1 ml.) and kept for 2–3 days at room temperature for dissolution before counting. A 0·4 ml. aliquot was used for radioactive assay.

Lung macrophages were recovered by two different methods. In the first experiment physiological saline (7 ml.) was injected slowly into the lungs as soon as they had been removed from the body. A thick needle was passed through the larynx into the trachea, saline was injected into each lobe and left for 10 seconds. Then the liquid was removed, the lungs were inverted in a Petri dish and the remainder of the saline was drawn up into a syringe (La Belle and Brieger, 1961). The cell suspension was centrifuged twice with saline for 15 min. in a MSE bench centrifuge, and the residue, after suspension in physiological saline (5 ml.) containing saponin (2 drops of 5% saponin per 25 ml. of saline) to destroy erythrocytes, was centrifuged again. This procedure was repeated twice. The cells were suspended in saline (5 ml.) and lung macrophages were counted by pipetting a small drop into a counting chamber (Mod-Fuchs Rosenthal, Depth 0·2 ml.) as used for white cells. The remaining cells were washed 4 times with saline by centrifugation. The residue was then suspended in saline (0·5 ml.) and tetraethylammonium hydroxide (0·5 ml.) was added. After standing overnight at room temperature 0·4 ml. aliquots were taken for radioactive assay.

In the second experiment the procedure was similar except that a different method was used for recovering macrophages from the lungs. The lungs were washed out with saline solution without removing them from the animal's body. 1·5 to 2·0 ml. saline being introduced into the bronchial tree via the larynx before the chest was opened. A further 5 ml. saline was introduced after removal of the lungs.

For counting radioactivity in blood, 1 ml. of heart blood was taken into a syringe containing 3·8% trisodium citrate (0·1 ml.) and 0·2 ml. was transferred into a stoppered test tube. Perchloric acid (0·2 ml.) was added and the contents were thoroughly mixed, and then 30% hydrogen peroxide (0·4 ml.) was added and the contents were again well mixed. The sample was placed in a water bath at 80° C. for 1 hour with occasional shaking (Mahin and Lofberg, 1966) and 0·4 ml. was taken for radioactive assay.

Twenty-four hour samples of faeces from each hamster were homogenized with distillated water (50 ml.) in an Ultra-Torrax homogenizer. 1 ml. of the homogenate was transferred to a stoppered test tube and 10 N sodium hydroxide (0·1 ml.) and hydrogen peroxide (0·1 ml.) were added. The samples were kept at 37° C. overnight before counting. Twenty-four hour samples of urine were diluted with distilled water (6 ml.) and 0·4 ml. aliquots used for counting (Rees, Rowland and Varcoe, 1966).

In all statistical calculations the Student " t " test was used.

### EXPERIMENTAL

### *Counting of the radioactivity in whole organs at various times*

*Experiment I.*—In the first experiment [$^3$H]BP with specific radioactivity 36 $\mu$Ci/mg. was used. 109 hamsters were divided into 3 groups:

*Group I.* 35 animals were injected intratracheally with [$^3$H]BP (5 mg.).

*Group II.* 47 animals were injected intratracheally with [$^3$H]BP (5 mg.) + crocidolite (1 mg.).

*Group III.* 27 hamsters were injected intratracheally with [$^3$H]BP (5 mg.) + carbon black (1 mg.).

In each case the material was injected as a suspension of aminosol vitrum (0·2 ml.).

Thirty-one animals died. Three hamsters from each group were killed at 3 hours, 6 hours, 1, 3, 7, 14, 21, 28, 35 days after the intratracheal injection and radioactivity remaining in lungs, livers and kidneys was estimated.

*Experiment II.*—In a repetition of the above experiment the specific activity of the [$^3$H]BP was 20 $\mu$Ci/mg., and animals weighing 130–150 g. were used. 70 animals were divided into 3 groups. Twenty-eight hamsters died, and the remaining 42 animals (Group I – 12; Group II – 16; Group III – 14) were killed (2 or 3 at each time) at 3, 7, 14, 21, 28 days after treatment.

*Recovery and counting of lung macrophages*

*Experiment III.*—[$^3$H]BP with specific activity 35·5 $\mu$Ci/mg. was used. Sixty-four hamsters were divided into three groups and treated as in Experiments I and II. Three hamsters from each group were killed 24 hours after injection and 2 hamsters from each group were killed 7, 14 and 21 days after injection.

Ten hamsters were used for counting the number of macrophages in normal lungs.

*Experiment IV.*—In this experiment 52 older hamsters were used. As in previous experiments they were divided into 3 groups. Three animals from each group were killed at 6 hours, 1, 7, 14, 21 days after treatment. In this experiment the total radioactivity present in the lungs after the recovery of macrophages was determined.

*Radioactivity present in blood, faeces and urine*

*Experiment V.*—Hamsters from Experiment III were used. One ml. of blood was taken from 3 animals on the 1st, 7th, 14th and 21st day of the experiment. Three hamsters from each group were placed in separate metabolic cages and 24-hour samples of faeces and urine were collected on the 3rd, 7th, 14th, 21st and 36th days after treatment.

### RESULTS

During the first hours after intratracheal injection of any of the test materials, the animals were distressed and severely dyspnoeic. In the groups injected with BP alone or BP + carbon black, these symptoms disappeared within 3 days, but in the group given BP + asbestos the condition of the hamsters deteriorated over a period of 5–10 days and several animals died. Thereafter the condition of survivors improved and the risk of death subsided. At autopsy, lobar and lobular pneumonia, mainly affecting the left lung, was found.

*Clearance of [$^3$H]BP from lungs*

The mean percentage of the total administered radioactivity in lungs at various times is shown in Table I and in Fig. 1 on a semilogarithmic scale.

The efficiency of clearance was striking in all 3 groups. Up to the 14th day only small differences between the 3 groups were apparent.

TABLE I.—*Percentage of Administered Radioactivity in Whole Lungs at Various Times*

| Group | Time | Exp. I | Exp. II | Exp. I and II together | Exp. IV* |
|---|---|---|---|---|---|
| $[^3H]$3,4-Benzopyrene alone | 3 hours | 46·3 ±2·13† | .. | 46·3 ±2·13 | 38·73±0·55 |
| | 6 hours | 22·63±0·16 | .. | 22·63±0·16 | 25·12±3·69 |
| | 1 day | 20·64±0·46 | .. | 20·64±0·46 | .. |
| | 3 days | 3·63±0·71 | 6·16±0·76 | 4·9 ±0·4 | .. |
| | 7 days | 3·27±0·005 | 1·29±0·098 | 2·28±0·18 | 1·16±0·12 |
| | 14 days | 1·05±0·01 | 0·28±0·008 | 0·67±0·07 | 0·27±0·01 |
| | 21 days | 0·05±0·007 | 0·12±0·017 | 0·094±0·01 | 0·106±0·005 |
| | 28 days | 0·07±0·007 | .. | 0·07±0·007 | .. |
| | 35 days | 0·04 | | 0·04 | .. |
| $[^3H]$3,4-Benzopyrene and asbestos | 3 hours | 52·51±0·35 | .. | 52·51±0·35 | 40·49±1·6 |
| | 6 hours | 37·78±0·41 | .. | 37·78±0·42 | 31·94±0·34 |
| | 1 day | 16·15±0·86 | .. | 16·15±0·86 | .. |
| | 3 days | 8·79±0·42 | 10·86±1·73 | 10·03±0·77 | .. |
| | 7 days | 1·01±0·09 | 2·02±0·5 | 1·61±0·25 | 1·04±0·08 |
| | 14 days | 0·36 | 0·41±0·06 | 0·40±0·034 | 0·34±0·05 |
| | 21 days | 0·24±0·02 | 0·3 ±0·017 | 0·27±0·009 | 0·23±0·01 |
| | 28 days | 0·31±0·06 | 0·25±0·01 | 0·27±0·01 | .. |
| | 35 days | 0·17±0·005 | .. | 0·17±0·005 | .. |
| $[^3H]$3,4-Benzopyrene and carbon black | 3 hours | 44·99±1·79 | .. | 44·99±1·79 | 45·00±0·65 |
| | 6 hours | 30·97±2·61 | .. | 30·97±2·61 | 25·30±1·44 |
| | 1 day | 14·21±1·32 | .. | 14·21±1·32 | .. |
| | 3 days | 1·63±0·23 | 3·97±0·18 | 3·03±0·27 | .. |
| | 7 days | 0·67±0·06 | 1·04±0·1 | 0·86±0·056 | 1·24±0·15 |
| | 14 days | 0·32±0·05 | 0·45±0·028 | 0·37±0·02 | 0·39±0·07 |
| | 21 days | 0·4 ±0·05 | 0·30±0·05 | 0·35±0·025 | 0·18±0·02 |
| | 28 days | 0·27±0·01 | 0·25±0·04 | 0·26±0·01 | .. |
| | 35 days | 0·25±0·02 | .. | 0·25±0·02 | .. |

* Total lung radioactivity after removal of lung macrophages.
† Standard error.

It is not certain whether these differences are real, though they were also seen in a further experiment not recorded in Table I: on the 3rd day after treatment an average of 13% of the radioactivity was found in the lungs of 3 hamsters given BP and asbestos compared with 5% following treatment with BP alone, and 6% following treatment with BP and carbon black.

The extent and consistency of the differences between the groups from the 21st day onwards suggests that they are real. After 21 days both asbestos and carbon black significantly increased the retention of radioactivity.

*The residual radioactivity in liver, kidneys and blood*

The mean percentage of the total radioactivity in liver kidneys and blood (1 ml.) are represented in Fig. 2, 3 and 4.

The amount of $[^3H]$BP and its metabolites in liver was similar in animals treated with BP + carbon black and BP + asbestos (Fig. 2). After BP alone less radioactivity was present in liver at all times after the 3rd day, although the differences between the groups were not large.

In the kidneys (Fig. 3) there was no difference during the first 21 days of the experiment, but after 28 days there was again less radioactivity in the group treated with BP alone.

FIG. 1.—Clearance of [$^3$H]3,4-benzopyrene from lung.

FIG. 2.—[$^3$H]3,4-benzopyrene in liver.

In blood (Fig. 4) radioactivity fell most quickly in the BP + carbon black-treated group whilst in animals treated with BP alone it remained high.

*Excretion of radioactivity in urine and faeces*

The mean percentages of the total administered radioactivity in 24-hour samples of urine and faeces are shown in Fig. 5 and 6.

FIG. 3.—[$^3$H]3,4-benzopyrene in kidneys.

FIG. 4.—Blood level of [$^3$H]3,4-benzopyrene.

The radioactivity in urine at various times after intratracheal injection was similar in all three groups of experimental animals (Fig. 5).

In faeces (Fig. 6) there appeared to be an earlier fall after treatment with BP + carbon black (between 14 and 21 days) than after treatment with BP alone (between 21 and 28 days).

FIG. 5.—Excretion of [$^3$H]3,4-benzopyrene in urine.

FIG. 6.—Excretion of [$^3$H]3,4-benzopyrene in faeces.

The total excretion of the label over 36 days in urine and faeces was similar, rather more being excreted in the faeces.

*Number and radioactivity of lung macrophages*

The results of these experiments are shown in Fig. 7 and 8.

In Fig. 7, the total numbers of macrophages recovered from the lungs at different times after treatment are represented on a semilogarithmic scale. More

FIG. 7.—Changes in lung macrophages. Each point represents mean value for 5 or 6 hamsters.

macrophages were recovered after the administration of BP + carbon black than after other treatments, and the numbers did not decrease greatly during the first 21 days of the experiments. Asbestos also increased the number of macrophages in comparison with the group given BP alone. At 21 days a mean of 2000 macrophages was obtained from the lungs after treatment with BP alone, 70,000 from BP + asbestos-treated animals, and 170,000 from BP + carbon black-treated animals.

Fig. 8 shows the radioactivity per macrophage plotted against time. At all times, this value was higher in the group treated with BP only, than in those given BP + asbestos or BP + carbon black. In Groups II and III, the radioactivity per macrophage was similar during the first week, but later it was higher in response to BP + carbon black than to BP + asbestos. This experiment was repeated and the differences were confirmed.

The total radioactivity of lung tissue after washing out macrophages is shown in Table I, as Experiment IV. The washing out of the lungs to obtain macrophages did not appear greatly to change the levels of residual radioactivity, which were essentially similar to those in the unwashed lungs in Experiments I and II.

FIG. 8.—Radioactivity per macrophage. Each point represents mean value for 5 or 6 hamsters.

### DISCUSSION

For practical purposes it is reasonable to assume that the pattern of distribution and elimination of the tritium label accurately reflects that of the BP molecule itself or its metabolites.

The rate of elimination of [$^3$H]BP like that of other materials (see Roe, 1968, for review) depends on several factors. The present results suggest that the period of clearance of material from the lung falls into 2 distinct phases, the first rapid and the second much slower. Much evidence in the literature both from studies on human lungs and from studies on animal lungs points to a similar conclusion (Green and Lane, 1964; Davies, 1949, 1952, 1961; La Belle and Brieger, 1959; Cember *et al.*, 1956; Hatch and Gross, 1964; and many others).

In the present experiments the initial, more rapid period of elimination lasted for about 2 weeks and was little influenced by the presence of the adsorbants, asbestos and carbon black. During that period 99% of label injected intratracheally was eliminated (Table I). The second, slow, period of elimination began between the second and the third weeks of the experiment. By that time it is probable that virtually all the free BP (i.e. unadsorbed or adsorbed on to eliminable dust particles) had been eliminated from the lung. As shown in Table I and Fig. 1, after 2 weeks there was a significant difference in the level of radioactivity in the lungs between groups of animals injected with BP alone and those injected with BP + asbestos or BP + carbon black. Pylev (1962, 1964) reported previously that the deposition and accumulation of carcinogen in lung tissue is important in relation to the induction of lung cancer. Although the residue of BP 2–3 weeks after a single intratracheal instillation is small—the level may be progressively raised by repeated instillations (Shabad, Pylev and Kolesnichenko, 1964). In this context, the threefold increase in the amount of the residue after a single instillation of BP with asbestos or carbon black may well be of considerable significance, particularly as the studies of radioactivity after washing out macrophages suggest that the BP-laden dust particles had penetrated into the lung substance. It is of interest that two compounds as dissimilar as asbestos and carbon black should have the same effect on retention of BP. The similarity of their effects suggests that it is simply their particulate nature combined with an ability to adsorb BP which prolonged the retention of the latter.

La Belle and Brieger (1959) attempted to estimate the number of lung macrophages in rats which had inhaled, or were injected intratracheally with, carbon black. They found that the rate of clearance of lung depended directly on macrophage activity. In the present experiments the instillation of BP + asbestos or BP + carbon black stimulated the macrophage activity more than BP alone, but the radioactivity per macrophage was less in the former than in the latter.

The levels of radioactivity in other organs and in blood, urine and faeces was similar irrespective of whether BP was given alone or mixed with a dust. The only constant difference observed was after the 14th day when the blood level in the BP + asbestos and BP + carbon black treated animals was lower than in hamsters given BP alone.

The results, like those of previous studies, suggest that the inhalation of insoluble particulate matter may be expected to enhance the carcinogenic effects of inhaled carcinogens such as BP. In connection with the cigarette smoking habit, this may mean that more attention should be paid than hitherto to the presence of chemically unreactive particulate matter in the smoke particularly to the presence of unburnt carbon particles. The results also suggest a mechanism whereby the inhalation of relatively inert dusts, such as asbestos, is associated with an increased risk of cancer development. Particles of asbestos in the lung tissue too large for macrophages to remove or below the bottom step of the mucus escalator, adsorb BP and other carcinogens from inhaled air or cigarette smoke and in doing so effectively prolong the residence of BP in the lungs. In this connection, a recent report by Selikoff and his colleagues (1968) is especially interesting. They found that the mortality from lung cancer among a group of asbestos workers was dependent upon, not only their exposure to asbestos dust, but also their smoking habits. In their study of 370 asbestos insulation workers, not one of 87 smokers died of bronchogenic carcinoma, whereas 24 out of 283 workers with a history of regular cigarette smoking died of the disease. Confirmation of this finding in relation to exposure to asbestos fibres, and possibly to other industrial dusts, is clearly an important growing point for further research.

### SUMMARY

A study in hamsters of the distribution and elimination of radioactivity after a single intratracheal instillation of a suspension of [$^3$H]3,4-benzopyrene (BP) in aminosol vitrum 10%, or of mixtures of BP and asbestos or of BP and carbon black in the same suspending medium, is reported. During the first 3 weeks of the experiment, radioactivity disappeared rapidly from the lungs irrespective of the presence or absence of carbon black. After 21 days, however, both asbestos and carbon black significantly increased retention of radioactivity. More macrophages could be recovered by use of a standard washing technique after the administration of BP + carbon black or BP + asbestos, than after the administration of BP alone, but the radioactivity per macrophage was highest in the group treated with benzopyrene only.

The levels of radioactivity found in liver, kidneys, blood and urine were similar in the 3 groups of animals. In the faeces there was an earlier fall of radioactivity after treatment with BP + carbon black (between 14 and 21 days) than after treatment with BP alone (between 21 and 28 days.

The significance of the results in relation to the causation of lung cancer in man is briefly discussed.

The work reported in this paper was undertaken during the tenure by one of us (L. Pylev) of a Research Training Fellowship awarded by the International Agency for Research on Cancer (Lyon, France). The authors are grateful to Professor Sir Alexander Haddow, F.R.S. for his interest in the work and to many members of the staff of the Chester Beatty Research Institute for their assistance or advice, particularly, Miss A. Albert, Mr. A. Coldman, Dr. P. Iveson, Mr. B. C. V. Mitchley and Dr. M. E. Whisson.

This investigation has been supported by grants to the Chester Beatty Research Institute (Institute of Cancer Research: Royal Cancer Hospital) from the Medical Research Council and the British Empire Cancer Campaign for Research, and by the Public Health Service Research Grant No. CA-03188 from the National Cancer Institute, U.S. Public Health Service.

### REFERENCES

BLACKLOCK, J. W. S.—(1957) *Br. J. Cancer*, **11**, 181.—(1961) *Br. J. Cancer*, **15**, 745.

CEMBER, H., HATCH, T. F., WATSON, J. A., GRUCCI, T. B. AND BELL, P.—(1956) *A.M.A. Archs ind. Hlth*, **13**, 170.

DAVIES, C. N.—(1949) *Br. J. ind. Med.*, **6**, 245.—(1952) *Br. J. ind. Med.*, **9**, 120.—(1961) *Ann. occup. Hyg.*, **3**, 219.

DELLA PORTA, G., KOLB, L. AND SHUBIK, P.—(1958) *Cancer Res.*, **18**, 592.

GREEN, H. L. AND LANE W. R. (1964) ' Particulate Clouds 1.' London (Spon).

HARINGTON, J. S.—(1962) *Nature, Lond.*, **193**, 43.—(1965) *Ann. N.Y. Acad. Sci.*, **132**, 31.

HARINGTON, J. S. AND ROE, F. J. C.—(1965) *Ann. N.Y. Acad. Sci.*, **132**, 439.

HATCH, T. F. AND GROSS, P.—(1964) ' Pulmonary Deposition and Retention of Inhaled Aerosols 1.' New York (Academic Press).

HERROLD, K. H. AND DUNHAM, L. J.—(1962) *J. natn. Cancer Inst.*, **28**, 467.—(1963) *Cancer Res.*, **23**, 773.

KUSCHNER, M., LASKIN, S., CRISTOFANO, E. AND NELSON, N. (1957) *Proc. 3rd natn. Cancer Conf.*, p. 485.

LA BELLE, C. W. AND BRIEGER, H.—(1959) *A.M.A. Archs ind. Hlth*, **20**, 100.—(1961) ' Inhaled Particles and Vapours.' *Proc. int. Symp.* organised by the *Br. occup. Hyg. Soc.*, p. 356.

MAHIN, D. T. AND LOFBERG, R. T.—(1966) *Analyt. Biochem.*, **16**, 500.

NISKANEN, K. O.—(1949) *Acta path. microbiol. scand.*, Suppl., 180.
PYLEV, L. N.—(1961) *Bull. exp. Biol. Med. U.S.S.R.*, **52**, 99.—(1962) *Vop. Onkol.*, **10**, 35.—(1963) *Proc. 8th int. Cancer Congr.* (Russian), **2**, 458.—(1964) *Vest. Akad. med. Nauk. SSSR*, **11**, 41.—(1967) *Higiena sanitaria* (Russian), **5**, 19.
REES, K. R., ROWLAND, G. F. AND VARCOE, J. S.—(1966) *Int. J. Cancer*, **1**, 197.
ROE, F. J. C.—(1968) 'Modern Trends in Toxicology.' London (Butterworths), p. 39.
ROE, F. J. C. AND WALTERS, M. A.—(1965) *Prog. exp. Tumor Res.*, **6**, 126.
ROE, F. J. C., WALTERS, M.A. AND HARINGTON, J. S.—(1966) *Int. J. Cancer*, **1**, 491.
SAFFIOTTI, U., BORG, S. A., GROFE, M. I. AND KARP, D. B.—(1964) *Chicago med. Sch. Q.*, **24**, 10.
SELIKOFF, I. J., HAMMOND, E. C. AND CHURG, J.—(1968) *J. Am. med. Ass.*, **204**, 106.
SHABAD, L. M., PYLEV, L. N. AND KOLESNICHENKO, T. S.—(1964) *J. natn. Cancer Inst.*, **33**, 135.
TIMBRELL, V. GILSON, J. C. AND WEBSTER, I.—(1968) *Int. J. Cancer*, **3**, 406.


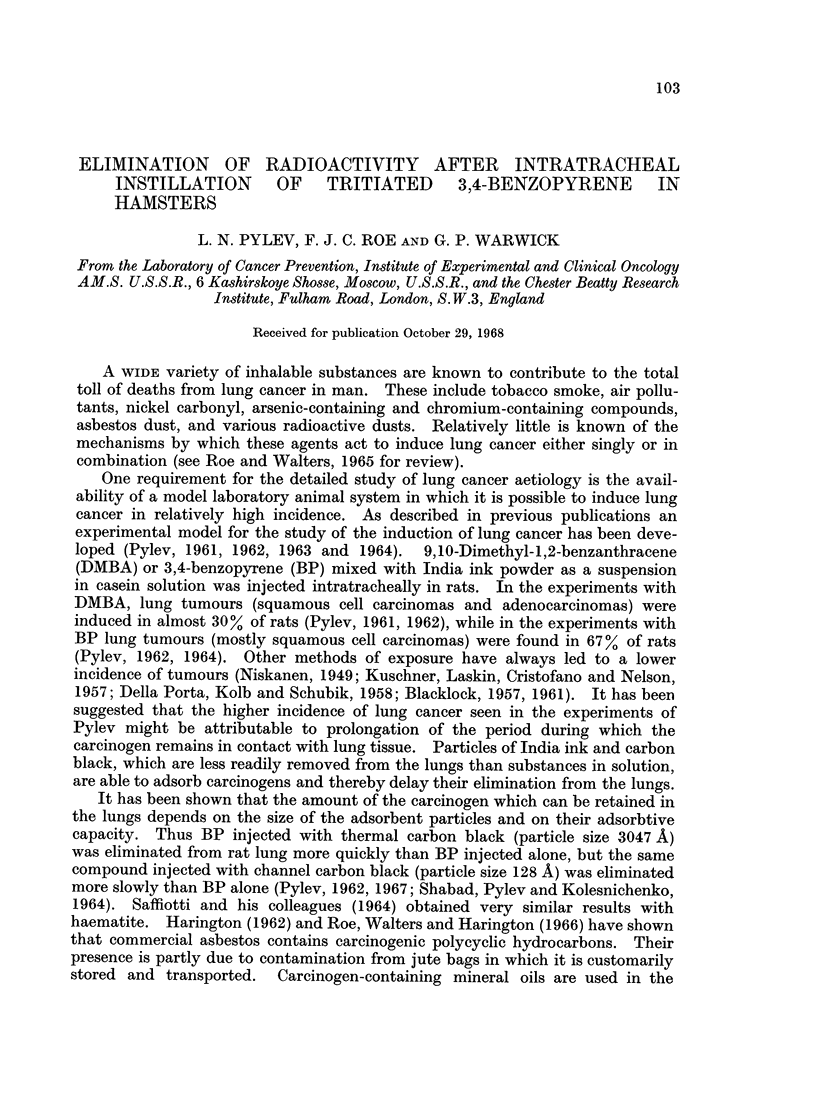

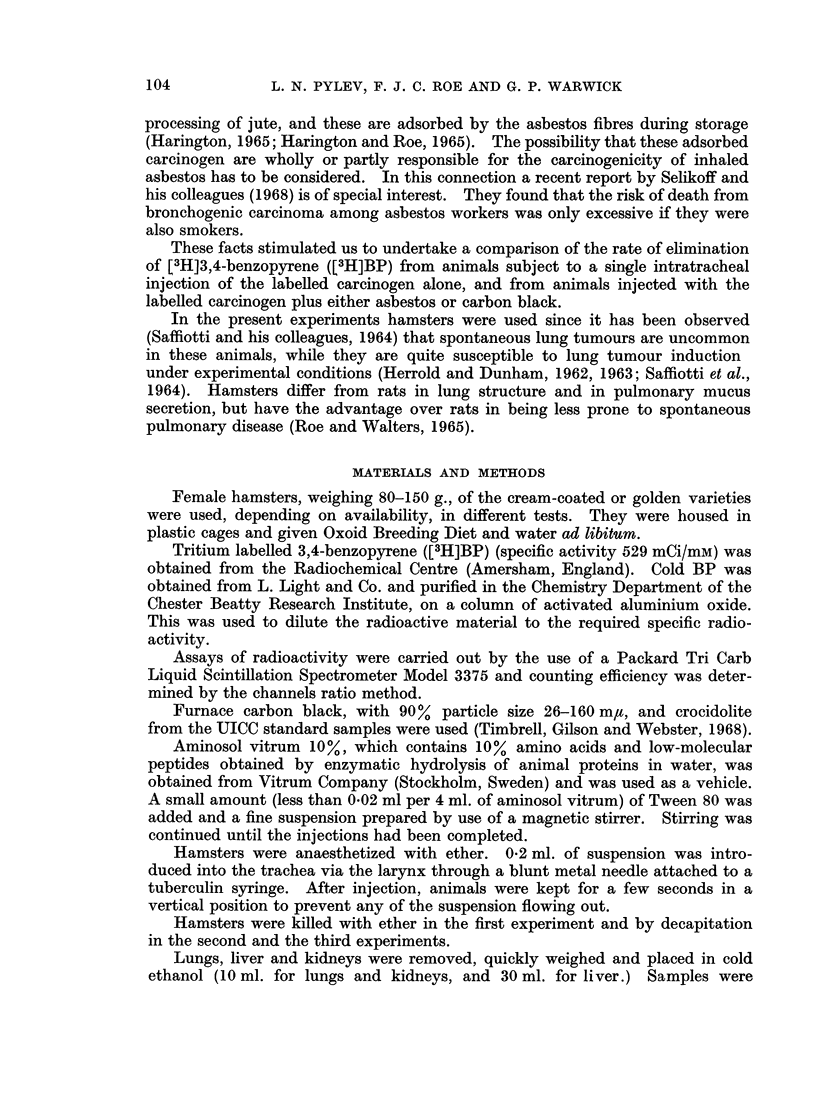

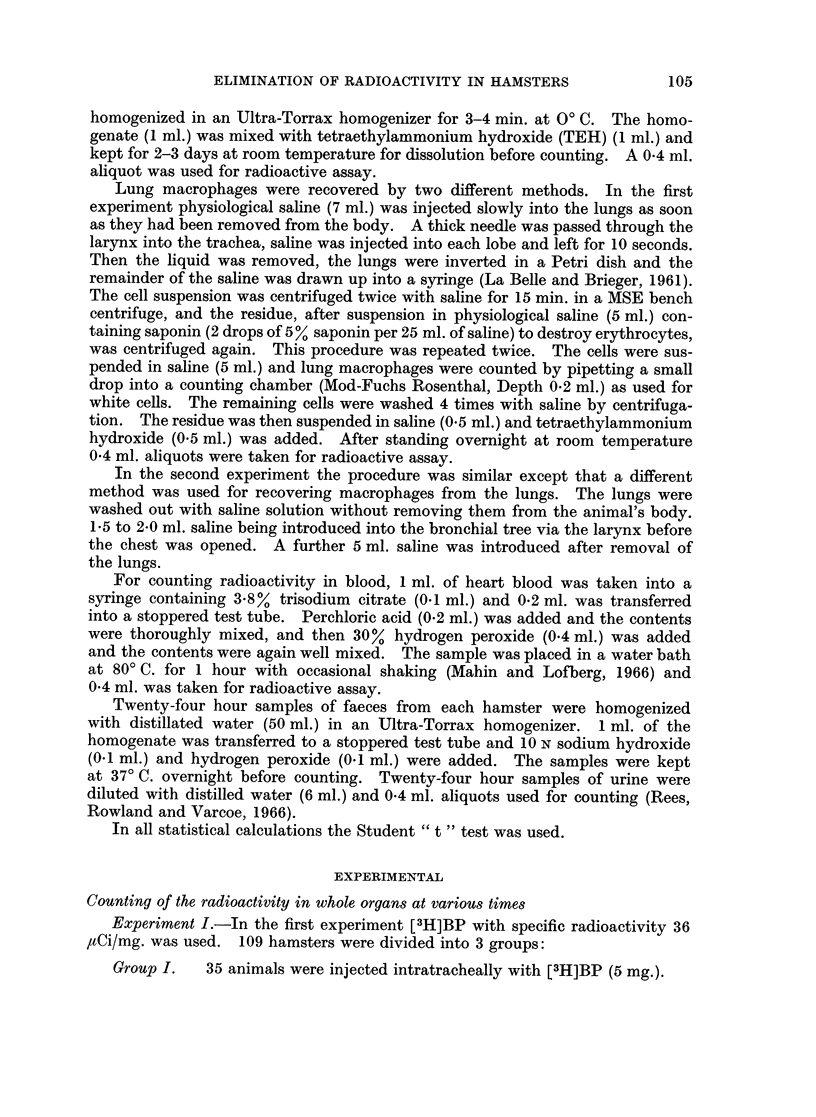

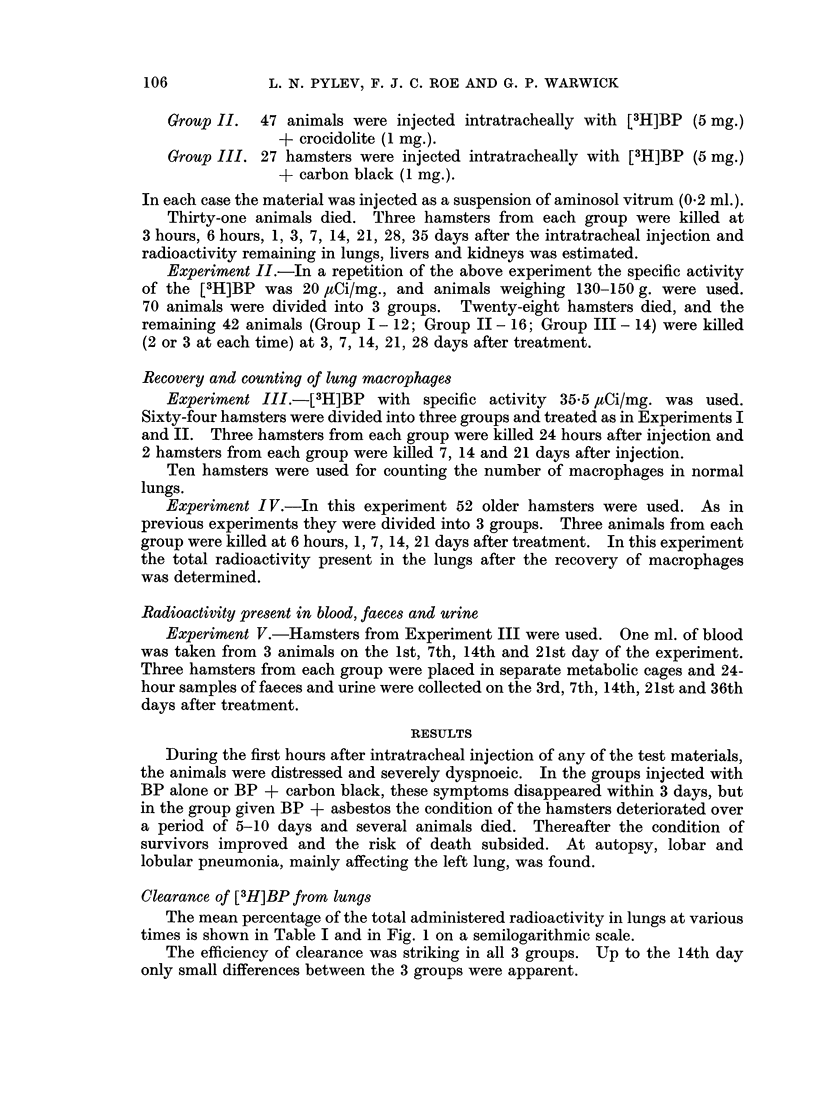

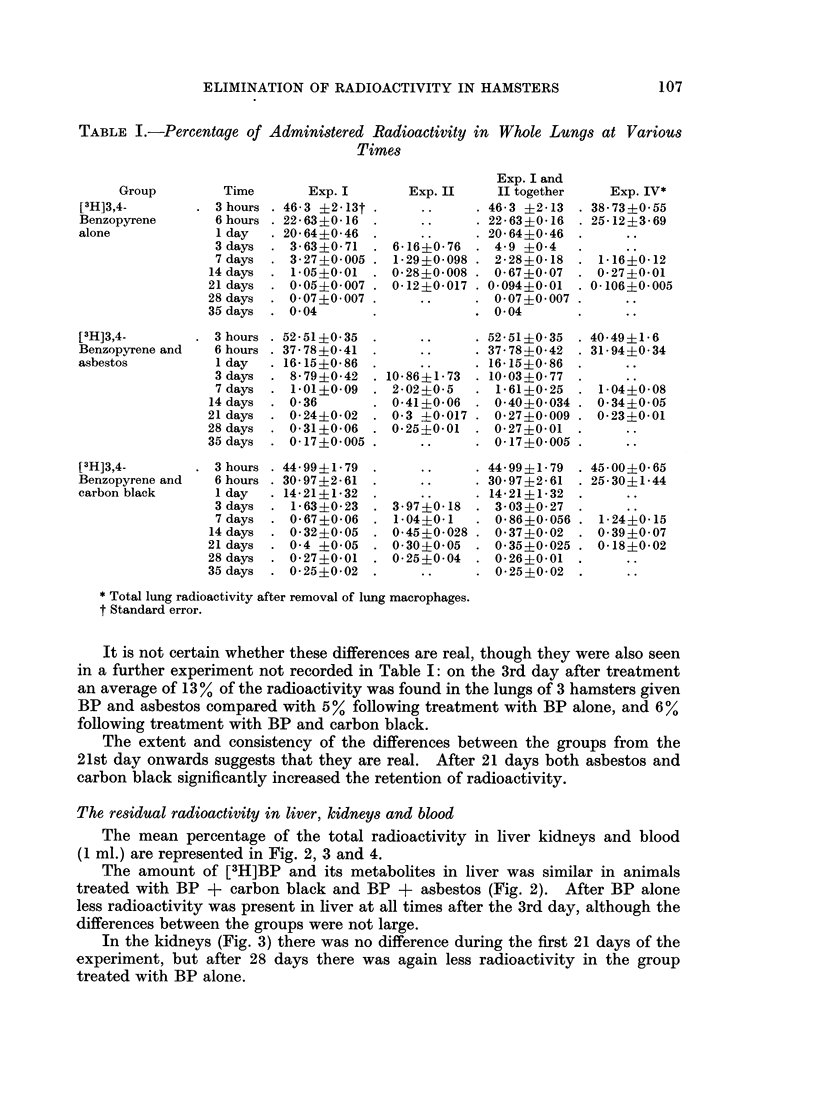

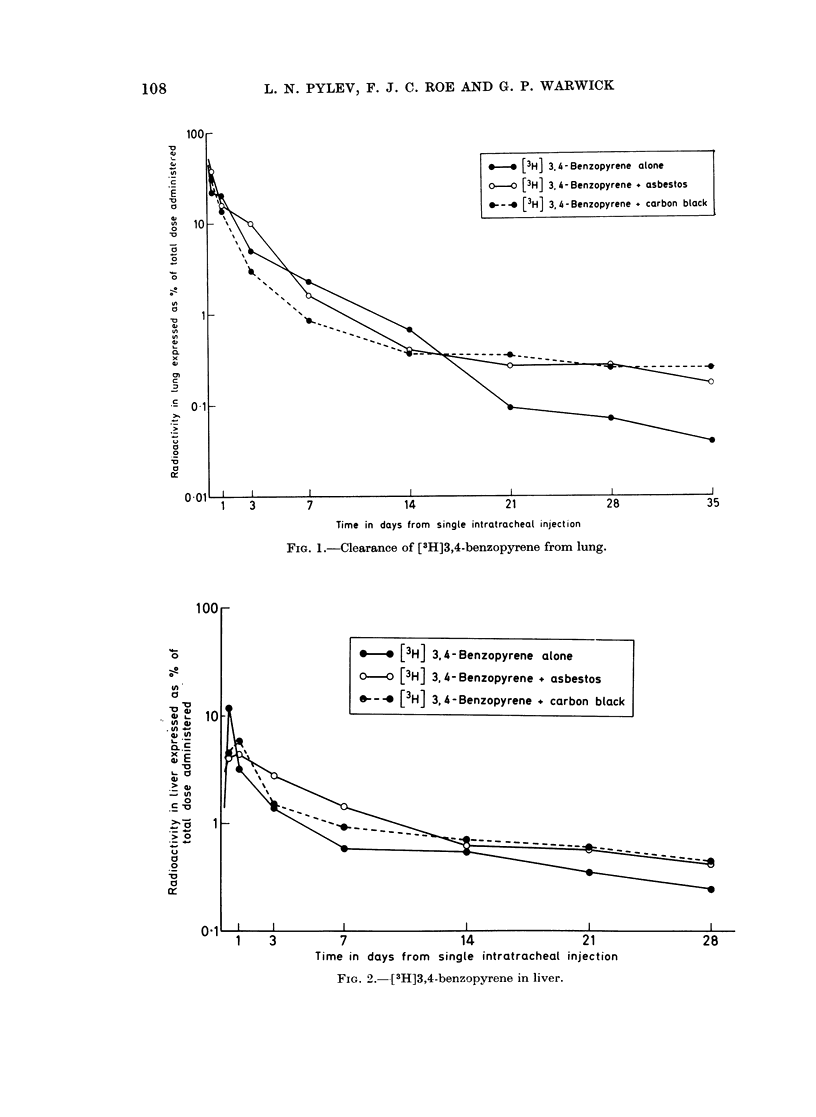

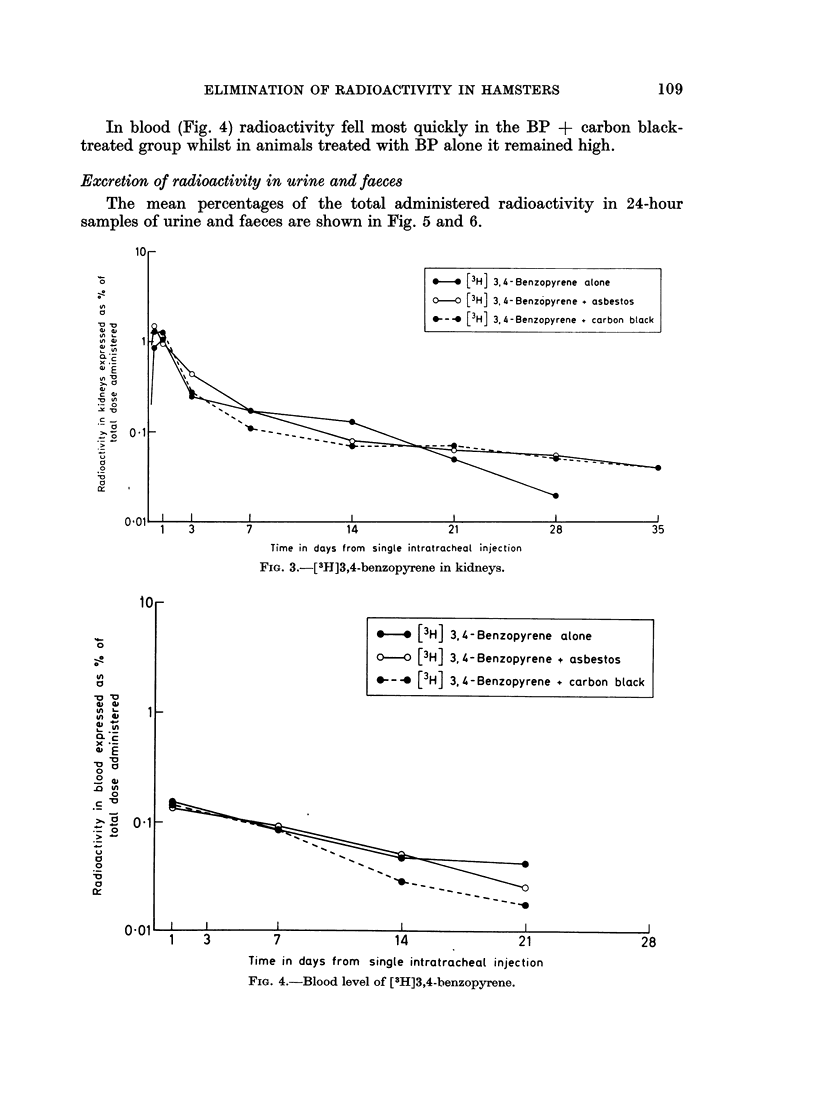

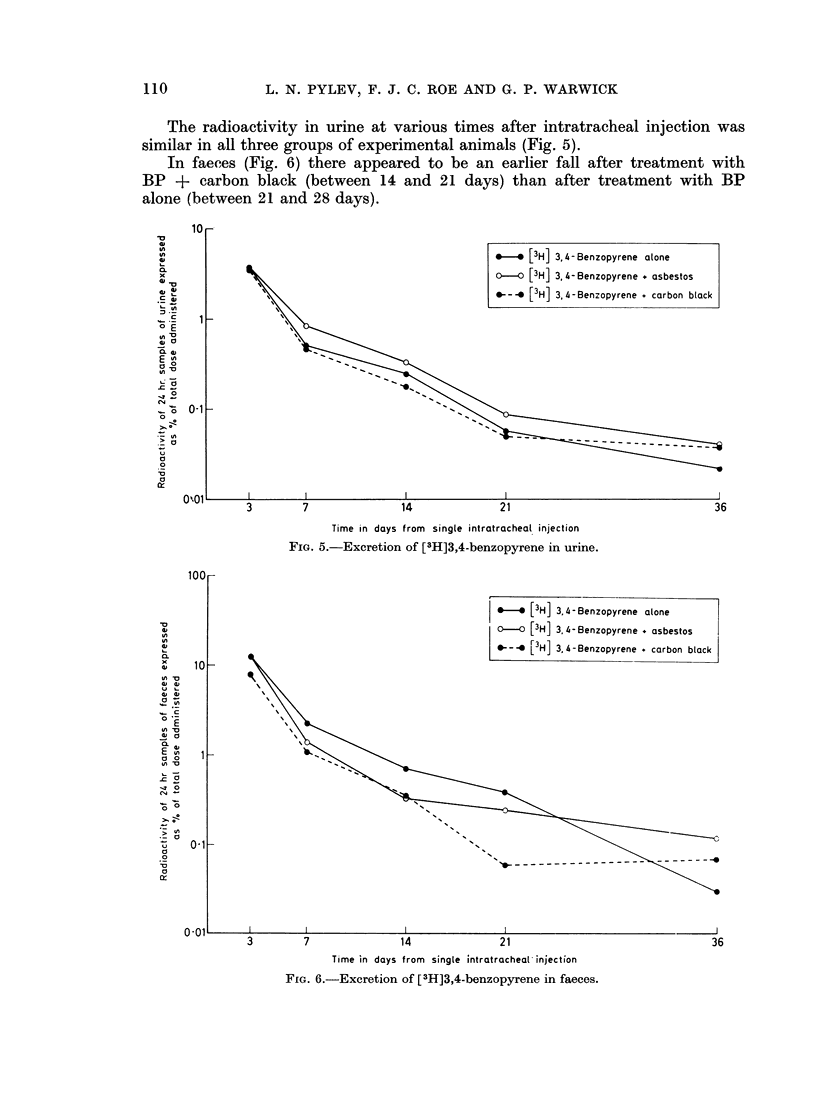

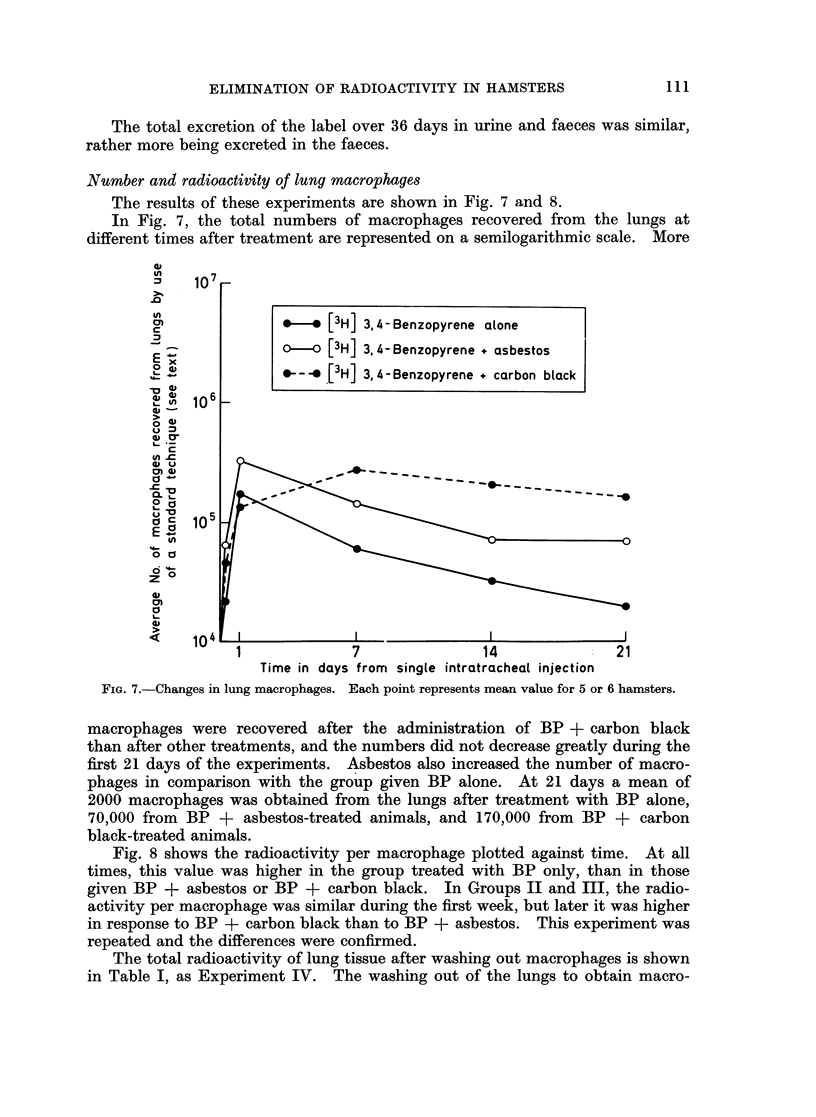

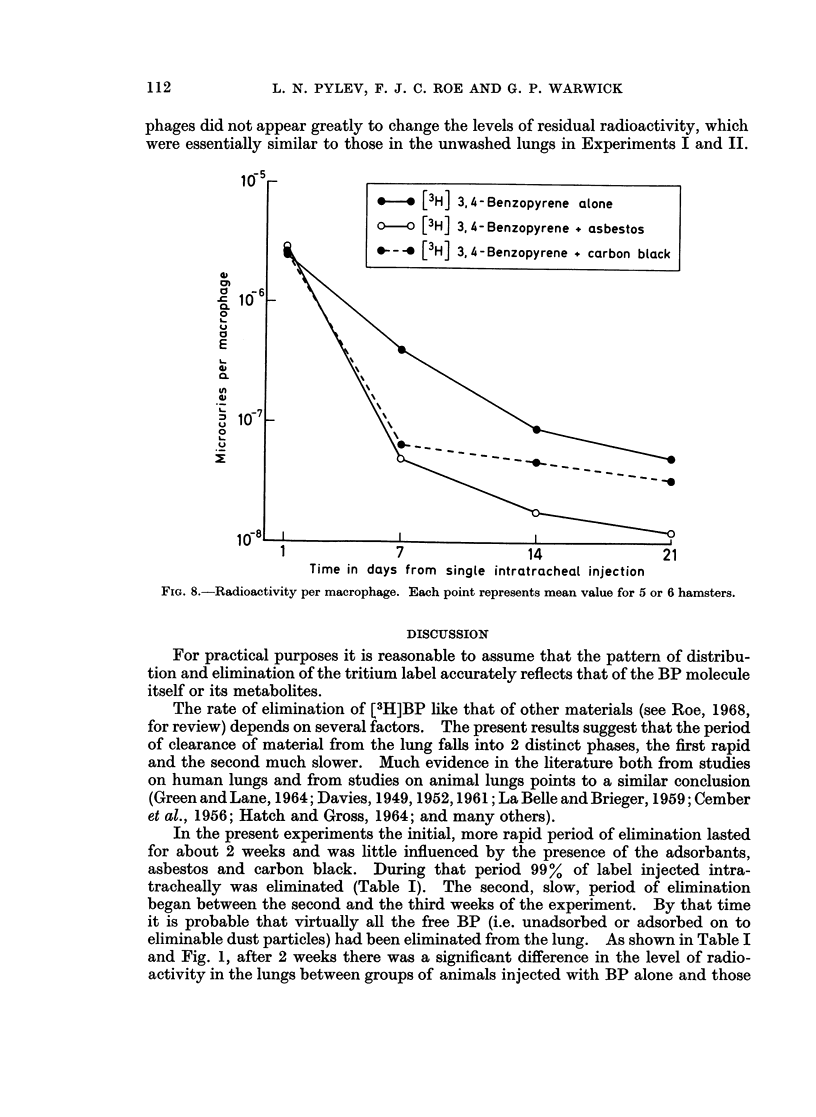

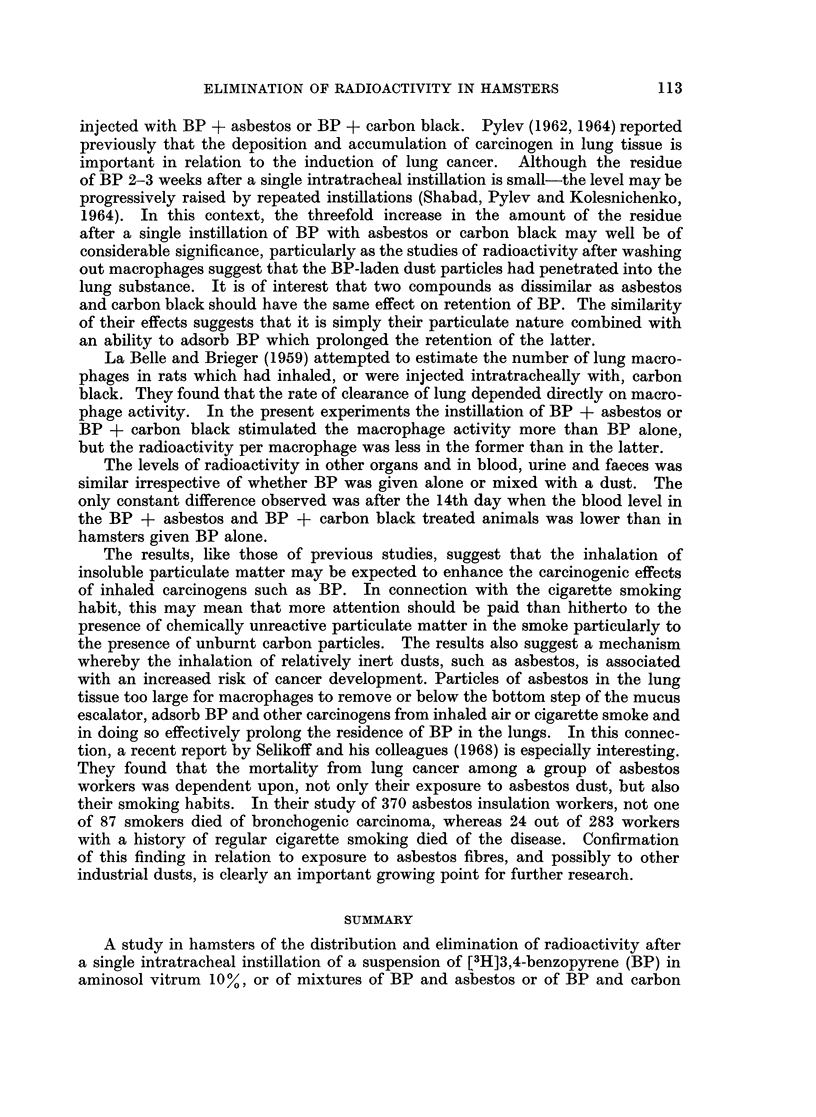

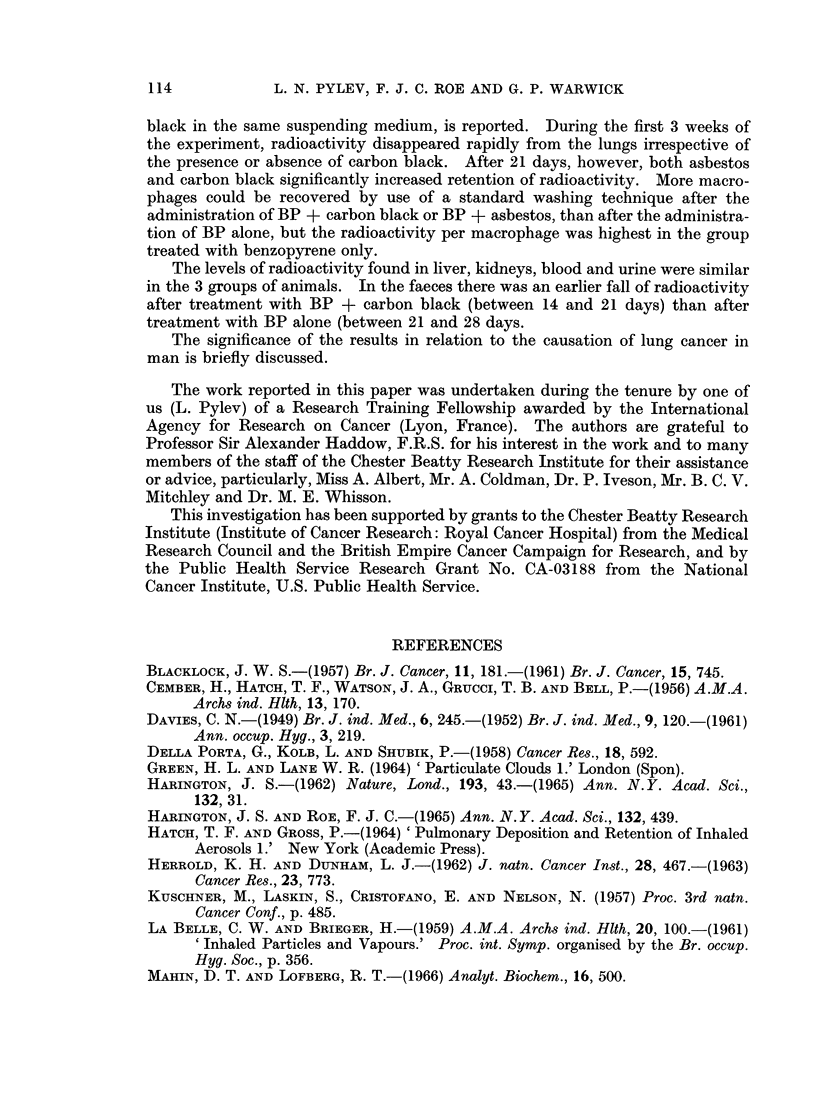

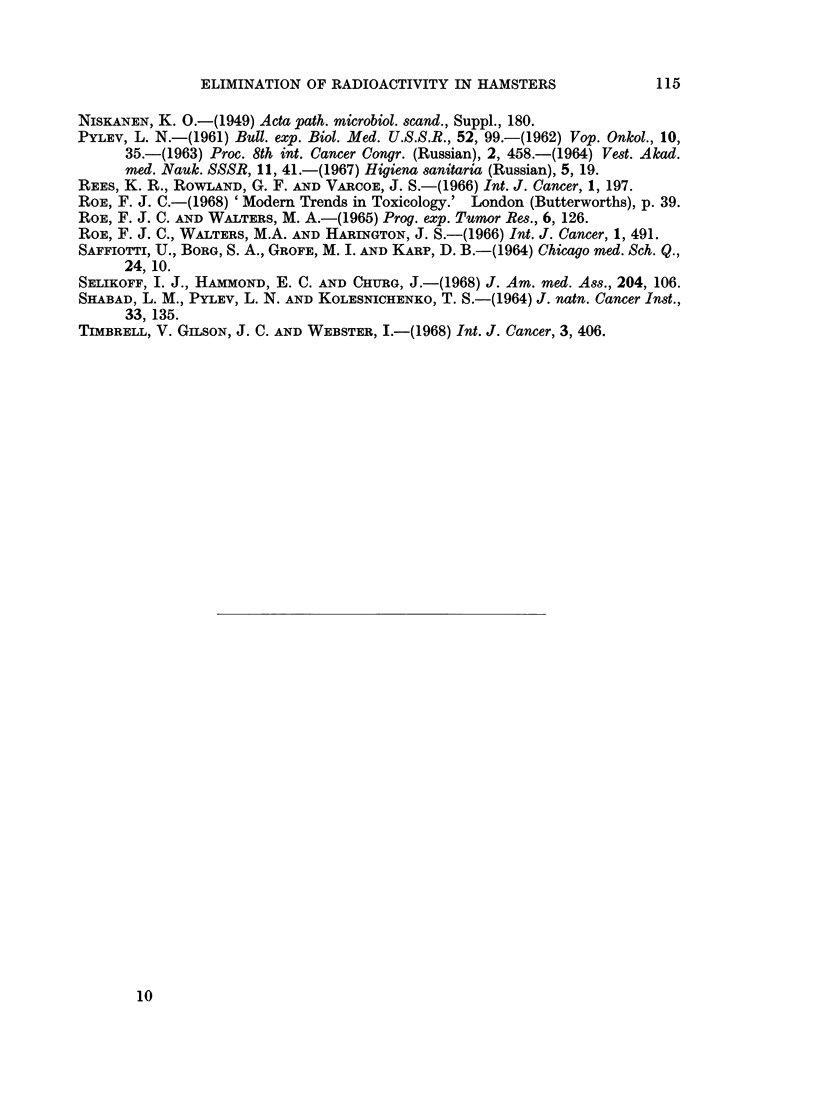


## References

[OCR_01014] BLACKLOCK J. W. (1961). An experimental study of the pathological effects of cigarette condensate in the lungs with special reference to carcinogenesis.. Br J Cancer.

[OCR_01016] CEMBER H., HATCH T. F., WATSON J. A., GRUCCI T., BELL P. (1956). The elimination of radioactive barium sulfate particles from the lung.. AMA Arch Ind Health.

[OCR_01020] DAVIES C. N. (1949). Inhalation risk and particle size in dust and mist.. Br J Ind Med.

[OCR_01024] DELLA PORTA G., KOLB L., SHUBIK P. (1958). Induction of tracheobronchial carcinomas in the Syrian golden hamster.. Cancer Res.

[OCR_01040] HERROLD K. M., DUNHAM L. J. (1962). Induction of carcinoma and papilloma of the Syrian hamster by intratracheal instillation of benzo[a]-pyrene.. J Natl Cancer Inst.

[OCR_01028] Harington J. S., Roe F. J. (1965). Studies of carcinogenesis of asbestos fibers and their natural oils.. Ann N Y Acad Sci.

[OCR_01067] Roe F. J., Walters M. A., Harington J. S. (1966). Tumour initiation by natural and contaminating asbestos oils.. Int J Cancer.

[OCR_01074] SHABAD L. M., PYLEV L. N., KOLESNICHENKO T. S. (1964). IMPORTANCE OF THE DEPOSITION OF CARCINOGENS FOR CANCER INDUCTION IN LUNG TISSUE.. J Natl Cancer Inst.

[OCR_01078] Timbrell V., Gibson J. C., Webster I. (1968). UICC standard reference samples of asbestos.. Int J Cancer.

